# Supplementary material for: Response-Surface-Methodology-Based Increasing of the Isotropic Thermal Conductivity of Polyethylene Composites Containing Multiple Fillers
Source: Polymers (Basel). 2022 Dec 22;15(1):39. doi: 10.3390/polym15010039 (PMC9824124; doi:10.3390/polym15010039)
Supplement: Supplementary file 1 [file polymers-15-00039-s001.zip › polymers-2080253-supplementary.pdf]

# Response surface methodology based increasing of the isotropic thermal conductivity of polyethylene composites containing multiple fillers – Supporting Information

## Table of contents

S1. SEM-images of the compositions ........ 1

S2. ANOVA Results ........ 3

S2.1. ANOVA Results for the TC FCCD ........ 3

S2.2. ANOVA Results for the melt viscosity FCCD ........ 4

S3. Determination of the dominant filler for the viscosity increase ........ 4

S4. Densities used of the polymer and fillers ........ 4

### S1. SEM-images of the compositions

**Run 2**

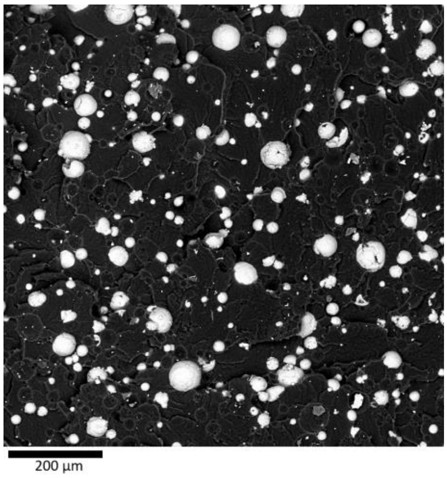


**Run 3**

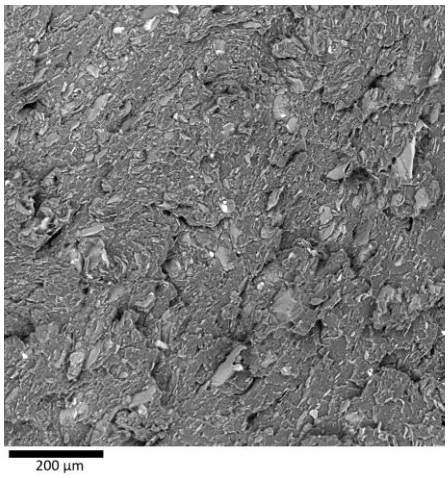


**Run 4**

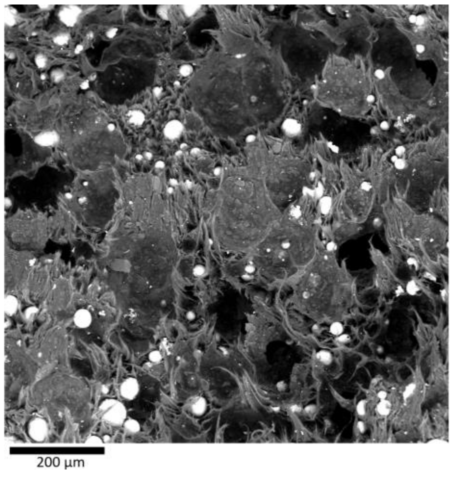


**Run 5**

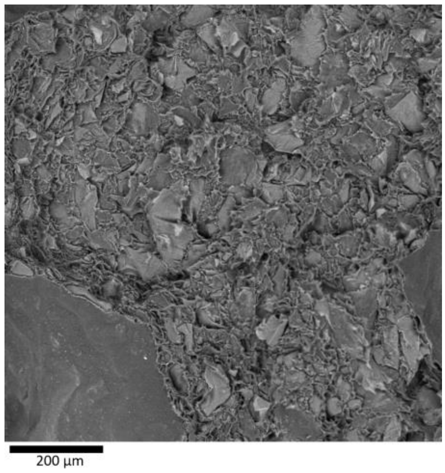

**Run 6**

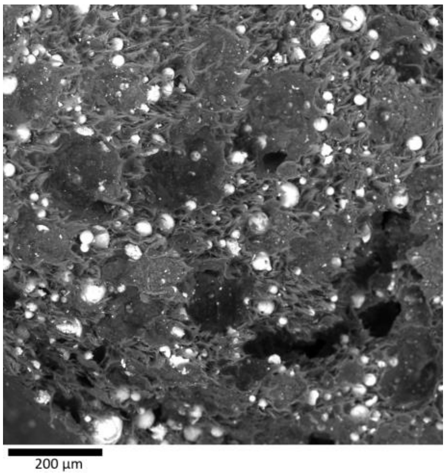


**Run 7**

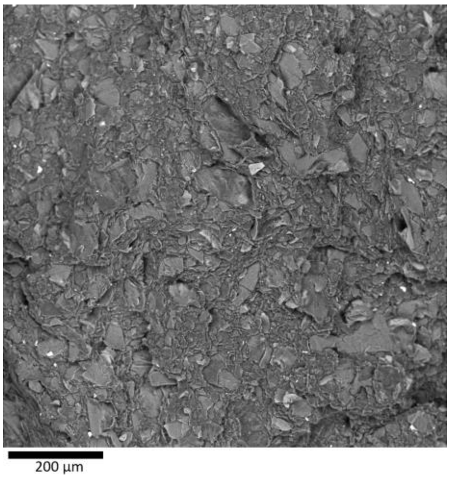


**Run 8**

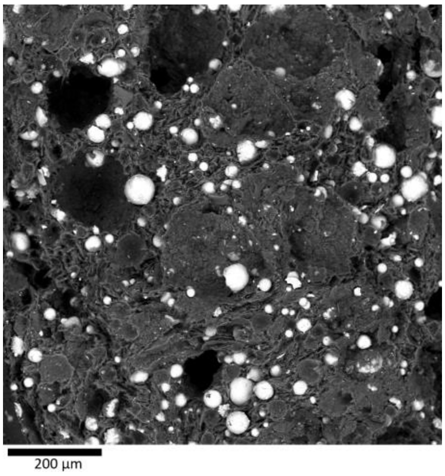


**Run 9**

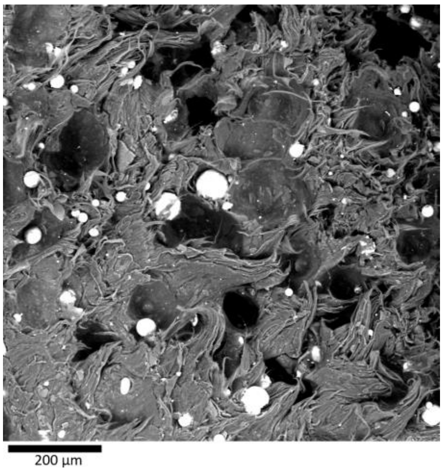


**Run 10**

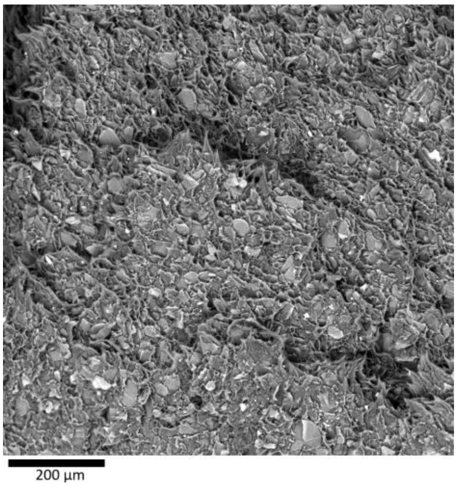


**Run 11**

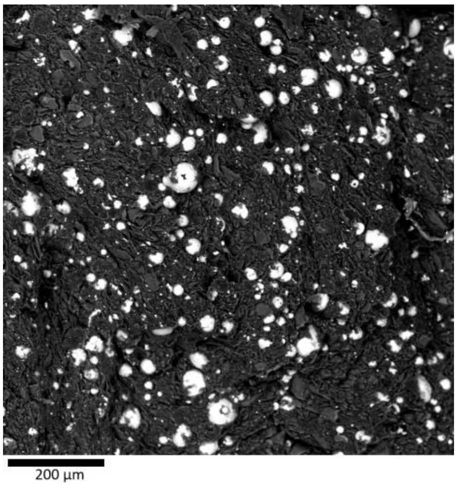

Run 12

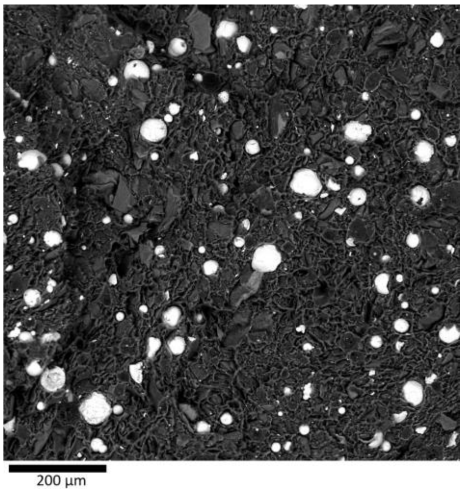


Run 13

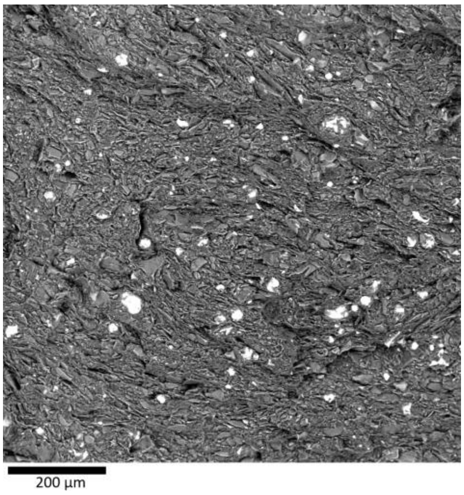


Run 14

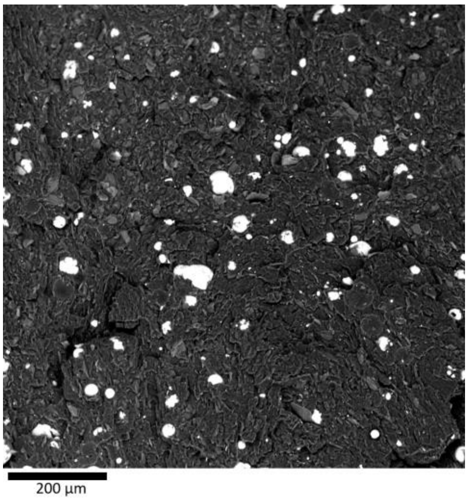


Run 15

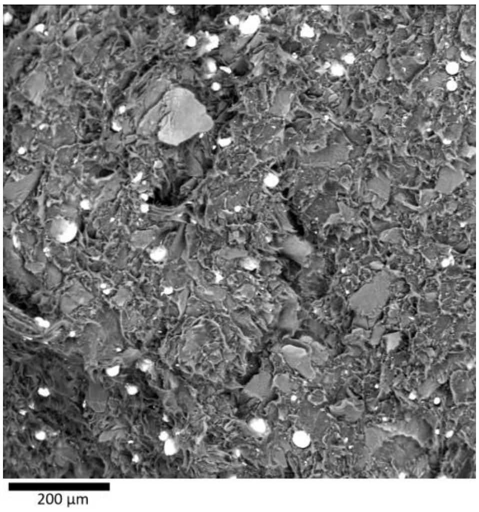


## S2. ANOVA Results

### *S2.1. ANOVA Results for the TC FCCD*

**Analysis of Variance**

| Source | DF | Sum of Squares | Mean Square | F Ratio |
|---|---|---|---|---|
| Model | 9 | 15.993274 | 1.77703 | 270.1134 |
| Error | 7 | 0.046052 | 0.00658 | Prob > F |
| C. Total | 16 | 16.039326 | | <.0001* |

**Parameter Estimates**

| Term | Estimate | Std Error | t Ratio | Prob>\|t\| |
|---|---|---|---|---|
| Intercept | 1.4016644 | 0.034707 | 40.39 | <.0001* |
| Alumina | 0.7081123 | 0.025649 | 27.61 | <.0001* |
| PP44 | 0.5796794 | 0.025649 | 22.60 | <.0001* |
| GFG75 | 0.7333924 | 0.025649 | 28.59 | <.0001* |
| Alumina*PP44 | 0.285701 | 0.028677 | 9.96 | <.0001* |
| Alumina*GFG75 | 0.3375624 | 0.028677 | 11.77 | <.0001* |
| PP44*GFG75 | 0.1667296 | 0.028677 | 5.81 | 0.0007* |
| Alumina*Alumina | 0.2156997 | 0.049553 | 4.35 | 0.0033* |
| PP44*PP44 | 0.0575522 | 0.049553 | 1.16 | 0.2835 |
| GFG75*GFG75 | 0.1186803 | 0.049553 | 2.40 | 0.0478* |

*S2.2. ANOVA Results for the melt viscosity FCCD*

**Analysis of Variance**

| Source | DF | Sum of Squares | Mean Square | F Ratio |
|---|---|---|---|---|
| Model | 9 | 254907123 | 28323014 | 45,3006 |
| Error | 7 | 4376571 | 625224,37 | **Prob > F** |
| C. Total | 16 | 259283693 | | <,0001* |

**Parameter Estimates**

| Term | Estimate | Std Error | t Ratio | Prob>\|t\| |
|---|---|---|---|---|
| Intercept | 12769,2 | 338,3455 | 37,74 | <,0001* |
| Al2O3 | 3820,01 | 250,0449 | 15,28 | <,0001* |
| PP44 | 937,79 | 250,0449 | 3,75 | 0,0072* |
| GFG75 | 2664,93 | 250,0449 | 10,66 | <,0001* |
| Al2O3*PP44 | 846,2 | 279,5587 | 3,03 | 0,0192* |
| Al2O3*GFG75 | 1480,55 | 279,5587 | 5,30 | 0,0011* |
| PP44*GFG75 | 256,45 | 279,5587 | 0,92 | 0,3895 |
| Al2O3*Al2O3 | 823,95 | 483,0718 | 1,71 | 0,1318 |
| PP44*PP44 | -675,25 | 483,0718 | -1,40 | 0,2049 |
| GFG75*GFG75 | 789,25 | 483,0718 | 1,63 | 0,1463 |

## S3. Determination of the dominant filler for the viscosity increase

| | $Al_2O_3$ | G | EG | Viscosity @ $\dot{\gamma} = 3\ s^{-1}$ (Pa.s) |
|---|---|---|---|---|
| m.% | 10 | 0 | 0 | |
| Coded level | -0,56 | 0 | 0 | 8337 |
| m% | 0 | 10 | 0 | |
| Coded level | 0 | 0,6 | 0 | 7820 |
| m% | 0 | 0 | 10 | |
| Coded level | 0 | 0 | 0,6 | 9568 |

## S4. Densities used of the polymer and fillers

| HDPE | G | EG | $Al_2O_3$ | CNT |
|---|---|---|---|---|
| 0.945 $g.cm^{-3}$ | 2.16 $g.cm^{-3}$ | 2.16 $g.cm^{-3}$ | 3.85 $g.cm^{-3}$ | 1.35 $g.cm^{-3}$ |
